# 6-Bromo-3-methyl-2-phenyl-3*H*-imidazo[4,5-*b*]pyridine

**DOI:** 10.1107/S1600536811022318

**Published:** 2011-06-18

**Authors:** Younes Ouzidan, El Mokhtar Essassi, Santiago V. Luis, Michael Bolte, Lahcen El Ammari

**Affiliations:** a Laboratoire de Chimie Organique Appliquée, Université Sidi Mohamed Ben Abdallah, Faculté des Sciences et Techniques, Route d’Immouzzer, BP 2202 Fès, Morocco; b Laboratoire de Chimie Organique Hétérocyclique URAC21, Faculté des Sciences, Université Mohammed V-Agdal, Avenue Ibn Battouta, BP 1014, Rabat, Morocco; c Departamento de Quimica Inorganica y Organica, ESTCE, Universitat Jaume I, E-12080 Castellon, Spain; d Institut für Anorganische Chemie, J. W. Goethe-Universität Frankfurt, Max-von-Laue-Strasse 7, 60438 Frankfurt/Main, Germany; e Laboratoire de Chimie du Solide Appliquée, Faculté des Sciences, Université Mohammed V-Agdal, Avenue Ibn Battouta, BP 1014, Rabat, Morocco

## Abstract

The two fused five- and six-membered rings building the mol­ecule of the title compound, C_13_H_10_BrN_3_, are approximately planar, the largest deviation from the mean plane being 0.004 (2) Å. The dihedral angle between the imidazo[4,5-*b*]pyridine mean plane and that of the phenyl ring is 41.84 (11)°. The structure is held together by slipped π–π stacking between symmetry-related mol­ecules, with an inter­planar distance of 3.583 (1) Å and a centroid–centroid vector of 3.670 (2) Å.

## Related literature

For background regarding biological activity of imidazo[4,5-*b*]pyridines, see: Cristalli *et al.* (1995 ▶); Bukowski & Kaliszan (1991 ▶); Aridoss *et al.* (2006 ▶); Bavetsias *et al.* (2007 ▶). For background to their pharmacological activity, see: Chen & Dost (1992 ▶); Weier *et al.* (1993 ▶).
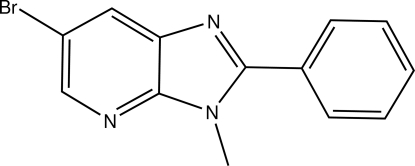


## Experimental

### 

#### Crystal data

C_13_H_10_BrN_3_
*M* *_r_* = 288.15
Orthorhombic, 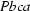

*a* = 13.7138 (4) Å
*b* = 6.7088 (2) Å
*c* = 25.3217 (7) Å
*V* = 2329.68 (12) Å^3^
*Z* = 8
Mo *K*α radiation
μ = 3.51 mm^−1^
*T* = 298 K
0.60 × 0.30 × 0.06 mm

#### Data collection

Bruker SMART CCD three-circle diffractometer
Absorption correction: multi-scan (*SADABS*; Bruker, 1997 ▶) *T*_min_ = 0.227, *T*_max_ = 0.825
13535 measured reflections
2378 independent reflections
1804 reflections with *I* > 2σ(*I*)
*R*_int_ = 0.047

#### Refinement

*R*[*F* ^2^ > 2σ(*F* ^2^)] = 0.033
*wR*(*F* ^2^) = 0.090
*S* = 1.04
2378 reflections
155 parameters
H-atom parameters constrained
Δρ_max_ = 0.37 e Å^−3^
Δρ_min_ = −0.40 e Å^−3^

### 

Data collection: *SMART* (Bruker, 1997 ▶); cell refinement: *SAINT* (Bruker, 1997 ▶); data reduction: *SAINT*; program(s) used to solve structure: *SHELXS97* (Sheldrick, 2008 ▶); program(s) used to refine structure: *SHELXL97* (Sheldrick, 2008 ▶); molecular graphics: *ORTEPIII* (Burnett & Johnson, 1996 ▶) and *ORTEP-3 for Windows* (Farrugia, 1997 ▶); software used to prepare material for publication: *WinGX* (Farrugia, 1999 ▶).

## Supplementary Material

Crystal structure: contains datablock(s) I, global. DOI: 10.1107/S1600536811022318/dn2697sup1.cif

Structure factors: contains datablock(s) I. DOI: 10.1107/S1600536811022318/dn2697Isup2.hkl

Supplementary material file. DOI: 10.1107/S1600536811022318/dn2697Isup3.cml

Additional supplementary materials:  crystallographic information; 3D view; checkCIF report

## Figures and Tables

**Fig. 1.**
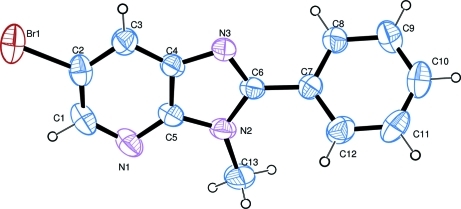
: Molecular view of the title compound with the atom-labelling scheme. Displacement ellipsoids are drawn at the 50% probability level. H atoms are represented as small circles of arbitrary radii.

## supplementary crystallographic information

### Comment

Heterocyclic ring systems having imidazo[4,5-*b*]pyridine nucleus can be considered as structural analogues of purines and have shown a diverse biological activity depending on the substituents of the heterocyclic ring. Their activity includes antiviral (Cristalli *et al.*, 1995), anticancer (Bavetsias *et al.*, 2007), tituberculostatic (Bukowski & Kaliszan, 1991) and antimitotic (Aridoss *et al.*, 2006) actions. They have also been evaluated as antagonists of various biological receptors including angiotensin-II (Chen & Dost, 1992) and platelet activating factor (PAF) (Weier *et al.*, 1993). Hence, the synthesis of imidazo[4,5-*b*]pyridine derivatives represents nowadays an important topic in organic synthesis.

The two fused five and six-membered rings are nearly planar with the maximum deviation of 0.004 (2) Å from N1. The dihedral angle between the imidazo[4,5-*b*]pyridine system and the phenyl ring is 41.84 (11)° (Fig. 1). The structure is held together by slipped π-π stacking between symmetry related molecules with interplanar distance of 3.583 (1) Å and centroid to centroid vector of 3.670 (2) Å resulting in a slippage of 0.79 Å.

### Experimental

To a solution of the 6-bromo-2-phenyl-1*H*-imidazo[4,5-*b*]pyridine (0.3 g, 1.09 mmol), potassium carbonate (0.2 g, 1.42 mmol) and tetra-n-butylammonium bromide (0.04 g, 0.1 mmol) in DMF (15 ml) was added methyl iodide (0.08 ml, 1.31 mmol). Stirring was continued at room temperature for 12 h. The salt was removed by filtration and the filtrate concentrated under reduced pressure. The residue was separated by chromatography on a column of silica gel with ethyl acetate/hexane (1/2) as eluent. The compound was recrystallized from ethanol.

### Refinement

H atoms were located in a difference map and treated as riding with C—H = 0.93 Å, and 0.96 Å for aromatic and methyl respectively and with *U*_iso_(H) = 1.2 *U*_eq_ (aromatic) and *U*_iso_(H) = 1.5 *U*_eq_(methyl).

### Crystal data

**Table:**

C_13_H_10_BrN_3_	*F*(000) = 1152
*M**_r_* = 288.15	*D*_x_ = 1.643 Mg m^−^^3^
Orthorhombic, *P**b**c**a*	Mo *K*α radiation, λ = 0.71073 Å
Hall symbol: -P 2ac 2ab	Cell parameters from 5157 reflections
*a* = 13.7138 (4) Å	θ = 3.0–29.6°
*b* = 6.7088 (2) Å	µ = 3.51 mm^−^^1^
*c* = 25.3217 (7) Å	*T* = 298 K
*V* = 2329.68 (12) Å^3^	Platelet, colourless
*Z* = 8	0.60 × 0.30 × 0.06 mm

### Data collection

**Table:**

Bruker CCD three-circle diffractometer	2378 independent reflections
Radiation source: fine-focus sealed tube	1804 reflections with *I* > 2σ(*I*)
graphite	*R*_int_ = 0.047
ω scans	θ_max_ = 26.4°, θ_min_ = 2.2°
Absorption correction: multi-scan (*SADABS*; Bruker, 1997)	*h* = 0→17
*T*_min_ = 0.227, *T*_max_ = 0.825	*k* = 0→8
13535 measured reflections	*l* = 0→31

### Refinement

**Table:**

Refinement on *F*^2^	Primary atom site location: structure-invariant direct methods
Least-squares matrix: full	Secondary atom site location: difference Fourier map
*R*[*F*^2^ > 2σ(*F*^2^)] = 0.033	Hydrogen site location: inferred from neighbouring sites
*wR*(*F*^2^) = 0.090	H-atom parameters constrained
*S* = 1.04	*w* = 1/[σ^2^(*F*_o_^2^) + (0.0361*P*)^2^ + 1.2509*P*] where *P* = (*F*_o_^2^ + 2*F*_c_^2^)/3
2378 reflections	(Δ/σ)_max_ = 0.002
155 parameters	Δρ_max_ = 0.37 e Å^−^^3^
0 restraints	Δρ_min_ = −0.40 e Å^−^^3^

### Special details

**Table:**

Geometry. All esds (except the esd in the dihedral angle between two l.s. planes) are estimated using the full covariance matrix. The cell esds are taken into account individually in the estimation of esds in distances, angles and torsion angles; correlations between esds in cell parameters are only used when they are defined by crystal symmetry. An approximate (isotropic) treatment of cell esds is used for estimating esds involving l.s. planes.
Refinement. Refinement of *F*^2^ against all reflections. The weighted *R*-factor *wR* and goodness of fit *S* are based on *F*^2^, conventional *R*-factors *R* are based on *F*, with *F* set to zero for negative *F*^2^. The threshold expression of *F*^2^ > σ(*F*^2^) is used only for calculating *R*-factors(gt) *etc*. and is not relevant to the choice of reflections for refinement. *R*-factors based on *F*^2^ are statistically about twice as large as those based on *F*, and *R*- factors based on all data will be even larger.

### Fractional atomic coordinates and isotropic or equivalent isotropic displacement parameters (Å^2^)

**Table:**

	*x*	*y*	*z*	*U*_iso_*/*U*_eq_	
Br1	0.65259 (2)	1.16657 (6)	0.626463 (10)	0.06788 (15)	
N1	0.60483 (17)	0.7251 (3)	0.51723 (8)	0.0509 (6)	
N2	0.60942 (14)	0.7570 (3)	0.42245 (7)	0.0396 (4)	
N3	0.64274 (15)	1.0833 (3)	0.41558 (8)	0.0425 (5)	
C1	0.6160 (2)	0.8347 (4)	0.56091 (10)	0.0528 (7)	
H1	0.6093	0.7719	0.5934	0.063*	
C2	0.63682 (17)	1.0361 (4)	0.56042 (9)	0.0477 (6)	
C3	0.64811 (18)	1.1434 (4)	0.51422 (10)	0.0464 (6)	
H3	0.6620	1.2790	0.5138	0.056*	
C4	0.63692 (16)	1.0313 (4)	0.46819 (9)	0.0385 (5)	
C5	0.61634 (17)	0.8291 (3)	0.47310 (9)	0.0386 (5)	
C6	0.62568 (16)	0.9162 (4)	0.38966 (9)	0.0369 (5)	
C7	0.62409 (16)	0.9055 (4)	0.33165 (9)	0.0378 (5)	
C8	0.58108 (18)	1.0590 (4)	0.30370 (9)	0.0463 (6)	
H8	0.5525	1.1644	0.3218	0.056*	
C9	0.5801 (2)	1.0574 (5)	0.24902 (10)	0.0561 (7)	
H9	0.5506	1.1610	0.2306	0.067*	
C10	0.6226 (2)	0.9037 (5)	0.22203 (11)	0.0590 (8)	
H10	0.6217	0.9025	0.1853	0.071*	
C11	0.6665 (2)	0.7515 (6)	0.24919 (12)	0.0641 (8)	
H11	0.6957	0.6476	0.2307	0.077*	
C12	0.66776 (19)	0.7512 (5)	0.30402 (11)	0.0531 (7)	
H12	0.6978	0.6475	0.3222	0.064*	
C13	0.5829 (2)	0.5528 (4)	0.40922 (12)	0.0607 (8)	
H13A	0.5461	0.5518	0.3770	0.091*	
H13B	0.5441	0.4973	0.4372	0.091*	
H13C	0.6409	0.4746	0.4048	0.091*	

### Atomic displacement parameters (Å^2^)

**Table:**

	*U*^11^	*U*^22^	*U*^33^	*U*^12^	*U*^13^	*U*^23^
Br1	0.0669 (2)	0.0970 (3)	0.03975 (18)	−0.00449 (16)	−0.00264 (12)	−0.01196 (14)
N1	0.0583 (14)	0.0484 (13)	0.0460 (12)	−0.0094 (11)	−0.0071 (10)	0.0159 (10)
N2	0.0467 (11)	0.0275 (10)	0.0445 (11)	−0.0009 (9)	−0.0056 (9)	0.0035 (9)
N3	0.0584 (13)	0.0312 (10)	0.0379 (10)	−0.0030 (9)	0.0000 (9)	0.0023 (9)
C1	0.0529 (15)	0.0641 (19)	0.0414 (14)	−0.0091 (14)	−0.0064 (11)	0.0176 (13)
C2	0.0411 (13)	0.0662 (19)	0.0358 (12)	−0.0012 (12)	−0.0042 (10)	0.0006 (12)
C3	0.0526 (15)	0.0436 (15)	0.0430 (13)	−0.0035 (11)	−0.0023 (11)	−0.0017 (11)
C4	0.0427 (13)	0.0344 (13)	0.0385 (12)	−0.0008 (10)	−0.0019 (9)	0.0043 (10)
C5	0.0385 (12)	0.0347 (12)	0.0426 (12)	−0.0022 (10)	−0.0061 (10)	0.0066 (10)
C6	0.0381 (12)	0.0316 (12)	0.0410 (12)	0.0030 (10)	−0.0028 (10)	0.0016 (10)
C7	0.0362 (11)	0.0391 (13)	0.0380 (12)	−0.0019 (10)	0.0003 (9)	−0.0014 (10)
C8	0.0522 (14)	0.0459 (15)	0.0408 (13)	0.0073 (12)	0.0051 (11)	0.0026 (11)
C9	0.0575 (16)	0.071 (2)	0.0402 (14)	0.0032 (14)	0.0022 (12)	0.0109 (13)
C10	0.0541 (15)	0.084 (2)	0.0389 (14)	−0.0069 (16)	0.0070 (12)	−0.0068 (14)
C11	0.0615 (19)	0.071 (2)	0.0594 (18)	0.0084 (16)	0.0145 (14)	−0.0249 (17)
C12	0.0514 (16)	0.0528 (16)	0.0551 (16)	0.0114 (13)	0.0018 (12)	−0.0064 (14)
C13	0.087 (2)	0.0329 (14)	0.0622 (17)	−0.0136 (14)	−0.0098 (15)	−0.0003 (13)

### Geometric parameters (Å, °)

**Table:**

Br1—C2	1.900 (3)	C7—C8	1.382 (3)
N1—C5	1.327 (3)	C7—C12	1.386 (4)
N1—C1	1.337 (3)	C8—C9	1.385 (3)
N2—C6	1.371 (3)	C8—H8	0.9300
N2—C5	1.374 (3)	C9—C10	1.367 (4)
N2—C13	1.456 (3)	C9—H9	0.9300
N3—C6	1.320 (3)	C10—C11	1.371 (5)
N3—C4	1.380 (3)	C10—H10	0.9300
C1—C2	1.381 (4)	C11—C12	1.388 (4)
C1—H1	0.9300	C11—H11	0.9300
C2—C3	1.382 (4)	C12—H12	0.9300
C3—C4	1.395 (3)	C13—H13A	0.9600
C3—H3	0.9300	C13—H13B	0.9600
C4—C5	1.391 (3)	C13—H13C	0.9600
C6—C7	1.471 (3)		
			
C5—N1—C1	113.2 (2)	C8—C7—C6	118.8 (2)
C6—N2—C5	106.23 (19)	C12—C7—C6	122.3 (2)
C6—N2—C13	129.4 (2)	C7—C8—C9	120.7 (3)
C5—N2—C13	124.3 (2)	C7—C8—H8	119.7
C6—N3—C4	104.8 (2)	C9—C8—H8	119.7
N1—C1—C2	123.7 (2)	C10—C9—C8	120.1 (3)
N1—C1—H1	118.2	C10—C9—H9	119.9
C2—C1—H1	118.2	C8—C9—H9	119.9
C1—C2—C3	122.7 (2)	C9—C10—C11	119.9 (3)
C1—C2—Br1	117.80 (19)	C9—C10—H10	120.1
C3—C2—Br1	119.5 (2)	C11—C10—H10	120.1
C2—C3—C4	114.5 (2)	C10—C11—C12	120.6 (3)
C2—C3—H3	122.8	C10—C11—H11	119.7
C4—C3—H3	122.8	C12—C11—H11	119.7
N3—C4—C5	110.2 (2)	C7—C12—C11	119.9 (3)
N3—C4—C3	131.6 (2)	C7—C12—H12	120.1
C5—C4—C3	118.2 (2)	C11—C12—H12	120.1
N1—C5—N2	126.4 (2)	N2—C13—H13A	109.5
N1—C5—C4	127.7 (2)	N2—C13—H13B	109.5
N2—C5—C4	105.9 (2)	H13A—C13—H13B	109.5
N3—C6—N2	112.9 (2)	N2—C13—H13C	109.5
N3—C6—C7	122.7 (2)	H13A—C13—H13C	109.5
N2—C6—C7	124.3 (2)	H13B—C13—H13C	109.5
C8—C7—C12	118.9 (2)		
